# Crack Segmentation Extraction and Parameter Calculation of Asphalt Pavement Based on Image Processing

**DOI:** 10.3390/s23229161

**Published:** 2023-11-14

**Authors:** Zhongbo Li, Chao Yin, Xixuan Zhang

**Affiliations:** School of Civil Engineering and Geomatics, Shandong University of Technology, Zibo 255049, China; 21507020778@stumail.sdut.edu.cn (Z.L.); zxxuan0209@163.com (X.Z.)

**Keywords:** pavement cracks, image processing, image enhancement, crack segmentation and extraction, calculation of crack parameters

## Abstract

Crack disease is one of the most serious and common diseases in road detection. Traditional manual methods for measuring crack detection can no longer meet the needs of road crack detection. In previous work, the authors proposed a crack detection method for asphalt pavements based on an improved YOLOv5s model, which is a better model for detecting various types of cracks in asphalt pavements. However, most of the current research on automatic pavement crack detection is still focused on crack identification and location stages, which contributes little to practical engineering applications. Based on the shortcomings of the above work, and in order to improve its contribution to practical engineering applications, this paper proposes a method for segmenting and analyzing asphalt pavement cracks and identifying parameters based on image processing. The first step is to extract the crack profile through image grayscale, histogram equalization, segmented linear transformation, median filtering, Sauvola binarization, and the connected domain threshold method. Then, the magnification between the pixel area and the actual area of the calibration object is calculated. The second step is to extract the skeleton from the crack profile images of asphalt pavement using the Zhang–Suen thinning algorithm, followed by removing the burrs of the crack skeleton image using the connected domain threshold method. The final step is to calculate physical parameters, such as the actual area, width, segments, and length of the crack with images obtained from the crack profile and skeleton. The results show that (1) the method of local thresholding and connected domain thresholding can completely filter noise regions under the premise of retaining detailed crack region information. (2) The Zhang–Suen iterative refinement algorithm is faster in extracting the crack skeleton of asphalt pavement, retaining the foreground features of the image better, while the connected-domain thresholding method is able to eliminate the missed isolated noise. (3) In comparison to the manual calibration method, the crack parameter calculation method proposed in this paper can better complete the calculation of crack length, width, and area within an allowable margin of error. On the basis of this research, a windowing system for asphalt pavement crack detection, WSPCD1.0, was developed. It integrates the research results from this paper, facilitating automated detection and parameter output for asphalt pavement cracks.

## 1. Introduction

Cracks are one of the most significant and common diseases in the early stages of road operation. The timely and accurate detection and scientific maintenance of cracks in existing and newly built roads are of cardinal importance for prolonging the service life of roads and reducing costs throughout their full lifecycle. Current research on pavement crack detection is still in the stage of identification and localization. Traditional methods of manual road disease detection are inefficient, dangerous, and block traffic; therefore, the authors proposed an asphalt pavement crack detection method based on an improved YOLOv5s model in their previous research. This method established an asphalt pavement crack dataset by capturing pavement images. They then trained and tested the model, revealing that the improved model outperformed other target detection models in each dataset.

With the development and improvement of deep learning technology, some scholars have suggested segmenting and extracting cracks through the training of deep learning models. For example, Chen et al. established an ACRP system based on close-range photogrammetry to digitize and calculate the parameters from acquired pavement texture images. Their results showed that the texture data obtained by the ACRP system had high accuracy and efficiency [1]. Yu et al. proposed a U-shaped encoder–decoder semantic segmentation network that combined Unet and Resnet. They introduced an attention module to improve detection results, adopted the Focus loss function to deal with the problem of unbalanced data categories in pavement crack segmentation, and achieved good segmentation results using a public crack dataset [2]. Liu et al. automatically detected and segmented cracks in asphalt pavements based on ground-penetrating radar (GPR) and convolutional neural network (R-CNN) with improved mask region, and established the relationship between actual crack area and the width of small cracks in GPR images [3]. Chen et al. proposed an unsupervised learning method based on outlier detection, which extracted the region of interest from the point cloud scene and isolated points within crack regions using an anomaly detection algorithm according to the characteristic distribution of points [4]. The fully supervised semantic segmentation method is based on deep learning and requires pixel-level annotations of the dataset during the model training stage, which can be complicated and time-consuming. The weakly supervised semantic segmentation method falls short of meeting the requirements in terms of annotation effect and detection accuracy, even though it allows for faster dataset annotations.

In recent years, the constant renewal and rapid development of image processing technology have led some scholars to propose image enhancement algorithms to eliminate crack image noise and highlight crack features, which could reduce the difficulties of crack segmentation. For example, Zhang et al. proposed an image enhancement algorithm based on automatic ridgelet transformation. The results showed that it could significantly enhance the overall and local contrast effect of road crack images [5]. Liu et al. proposed a multiscale Retinex image enhancement algorithm incorporating wavelet transformation. The results showed that this algorithm could effectively remove image shadows and enhance crack features [6]. Yang et al. proposed a three-stage asphalt pavement crack location and segmentation method combining traditional digital image processing technology and deep learning methods. The first step was to preprocess the asphalt pavement crack image using Guided filtering and the Retinex method. The second step was to locate asphalt pavement cracks using the YOLO-SAMT object detection model. The final step was to extract cracks using the K-means clustering algorithm. The results showed that it effectively improved crack profile extraction accuracy [7]. Vivekananthan et al. used the grayscale discriminant method to preprocess the image, set the threshold using the Otsu algorithm, and used the Sobel filter to detect cracks on the edges of the image pixel. Their results showed that the accuracy of this crack detection method was up to 95% [8]. Li et al. described an innovative vision-based pavement crack detection strategy. They proposed an ellipse fitting method to compute mesh crack blocks, and the results demonstrated the potential of this innovative deep learning approach for automated pavement quality index computation [9].

The evaluation of the damage degree index for cracks is reflected in the pavement crack damage rate. However, in most cases, deep learning or image processing only contributes to the identification and location of pavement cracks, making little contribution to practical engineering. Therefore, this paper proposes a method based on image processing for the segmentation extraction and parameter calculation of asphalt pavement cracks based on the improved model proposed in the previous study, which eliminates the crack image noise, extracts the crack contour image and the skeleton image through the image enhancement technique, and calculates the physical parameters of cracks according to the extracted image of the cracks. Meanwhile, on the basis of the above study, the windowing system of asphalt pavement crack detection WSPCD1.0 is developed to realize the automation of the cracks detection of the asphalt pavements and the parameter outputs and contribute to the crack detection work.

## 2. Image Enhancement Method

### 2.1. Crack Image Dataset

**Figure 1:**
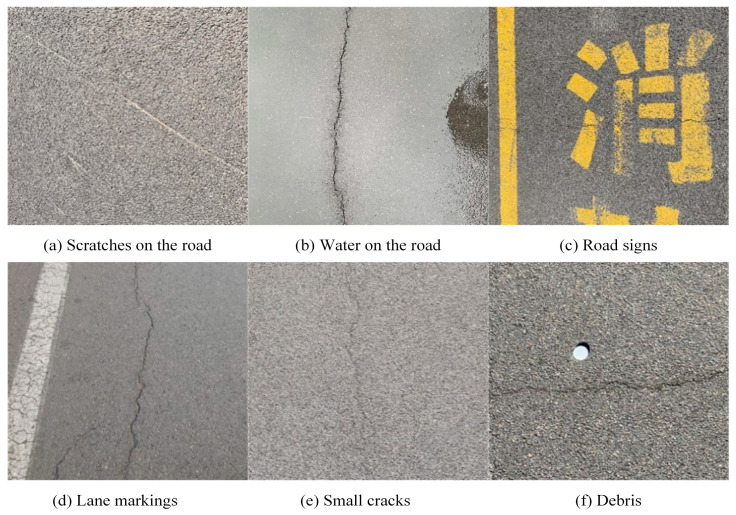
An example of detailed photography. (**a**) Scratches on the road. (**b**) Water on the road. (**c**) Road signs. (**d**) Lane markings. (**e**) Small cracks. (**f**) Debris.

The crack images used in this paper were obtained from the CFD dataset and the self-collected dataset [10] of some roads in Zhangdian District, Zibo City, Shandong Province. In order to improve the richness of the self-acquired datasets and targets, and to improve the detection capability of the model in a certain aspect, this paper captures the complex pavement environment appearing in asphalt pavement, including lane markings, shadows, glare, debris, lane lines, water on the road surface, etc. As shown in Figure 1, the model can avoid misjudging the pavement scratches, lane markings, etc., in the image. Images depicting cracks with tree branch shadows can significantly enhance the accuracy of the model in detecting cracks on the road surface under shadow; furthermore, images exhibiting cracks with strong exposures taken on sunny days and images of cracks with weak exposures captured on cloudy days can greatly enhance the accuracy of the model in identifying cracks on the road surface under various weather and lighting conditions. Experiments have proved that this method can effectively improve the generalization ability of the model, the accuracy of the model in identifying multiple cracks under different weather conditions, and the accuracy of the model in identifying multiple cracks.

**Figure 2:**
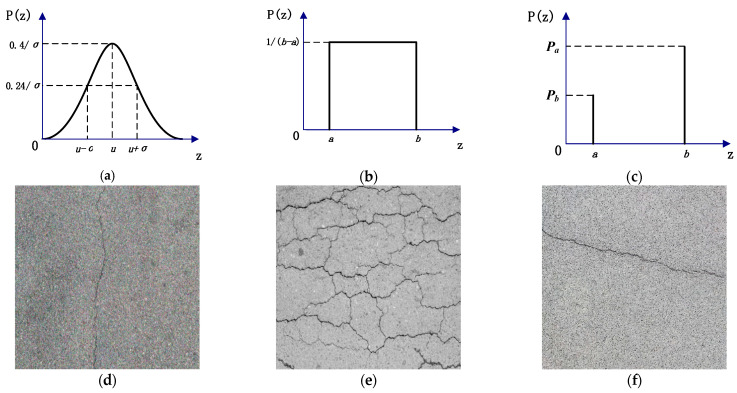
Images of various noises. (**a**) Gaussian noise probability density function. (**b**) Uniform noise probability density function. (**c**) Impulse noise probability density function. (**d**) Gaussian noise effect. (**e**) Uniform noise effect. (**f**) Impulse noise effect.

As the image acquisition process is easily affected by various factors such as light, equipment, and acquisition methods, the resulting pavement crack image may contain Gaussian, uniform, and salt-and-pepper (impulse) noise [11]. Confronted with small cracks or complex pavement environments, the probability density functions of various types of noise are shown in Figure 2. These noises will cause the feature and detail information of the crack to not be prominent enough, resulting in the information of the background region being stronger than the information of the crack region, thus affecting the effectiveness of the crack extraction. In addition, due to the large asphalt aggregate gap in asphalt pavement, if the grayscale value of the asphalt aggregate gaps is close to the value of the cracks in the pavement, it will cause great interference in the segmentation and extraction of the cracks in the image processing process.

To solve the problems of low resolution and various noises in crack images, this paper adopts the image enhancement method [12] to improve the quality of crack images. It improves the effect of crack segmentation and extraction by highlighting the features and details of the intercrack region while weakening the background of uninteresting regions. At present, there are two types of common image enhancement methods: the enhancement methods based on the spatial domain (space domain) [13,14,15], which primarily include point pixel processing and area processing; the enhancement methods based on the frequency domain (frequency domain) [16,17,18], which mainly include low-pass filtering, high-pass filtering, homomorphic filtering, etc. This paper focuses on the image enhancement method based on the spatial domain.

### 2.2. Image Grayscale Processing

**Figure 3:**
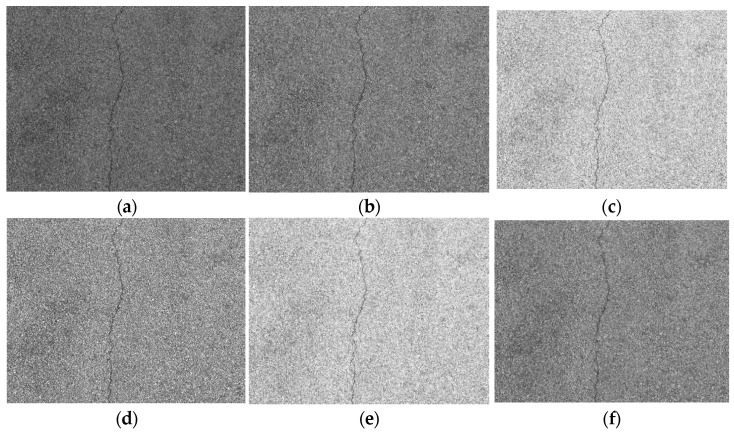
Results of image grayscale processing. (**a**) Component method *f* = *R*. (**b**) Component method *f* = *G*. (**c**) Component method *f* = *B.* (**d**) Average value method. (**e**) Maximum value method. (**f**) Weighted average method.

The commonly used methods for selecting the new component *f* of the image grayscale processing are the component method *f* = *R* or *f* = *G* or *f* = *B*, the average value method *f* = (*R* + *G* + *B*)/3, the maximum value method *f* = max (*R*, *G*, *B*), and the weighted average method *f* = 0.30*R* + 0.59*G* + 0.11*B* [19]. Figure 3 shows the grayscale processing results of these four methods. In this paper, the average value method is used. It gives better visual results and more balanced grayscale processing results.

### 2.3. Histogram Equalization

**Figure 4:**
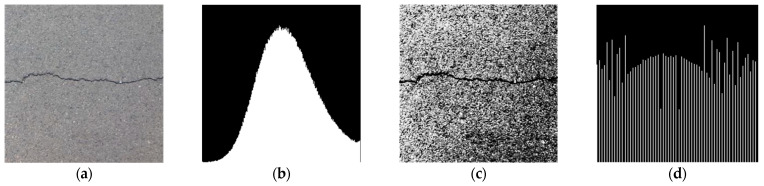
Results of histogram equalization. (**a**) Original grayscale image. (**b**) Original image histogram. (**c**) Equalized grayscale image. (**d**) Equalized histogram.

Histogram equalization is used to change the grayscale value of the image to a form that is evenly distributed throughout the grayscale range. This enhances the contrast of the image by adjusting the dynamic range of the grey values [20]. Figure 4 shows the results of the image processing. In the original image (a), there is a certain contrast between the background area and the crack area. However, affected by various interferences such as light and noise, the image is blurred and distorted, losing most of the details of the crack profile. In addition, the grayscale value of each pixel in the original image histogram (b) is extremely unevenly distributed. A single peak is clearly visible. In the grayscale image (c) after histogram equalization, the contrast between the background area and the crack area is significantly enhanced. The crack texture and profile are clearly visible and the image is rich in detail. And in the equalized histogram (d), the gray level of each pixel is relatively evenly distributed, which enhances the quality of the image.

### 2.4. Image Grayscale Transformation

Grayscale transformation changes the grayscale value of each pixel according to certain rules. This changes the grayscale range of the image and makes the visual effect of the image clearer [21]. It mainly includes the grayscale linear transformation method [22], the grayscale segmented linear transformation method [23], and the grayscale nonlinear transformation method [24].

(1) Grayscale Linear Transformation Method

**Figure 5:**
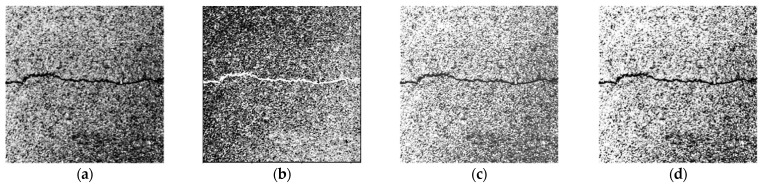
Image grayscale linear transformation. (**a**) Original image. (**b**) Image after grayscale inversion. (**c**) Increased grayscale. (**d**) Increased contrast.

The image grayscale linear transformation method includes image grayscale inversion, image grayscale adjustment, and image contrast adjustment. Figure 5 shows the processing results of the three grayscale linear transformation methods on the histogram equalized image. In the figure, (b) is the effect diagram of image inversion, which converts the original pixel with a larger grayscale value to a smaller one, and also converts the original pixel with a smaller gray value to a larger one; (c) is the result of image grayscale adjustment, which increases the grayscale value of each pixel in the image by 50 (after the grayscale adjustment, the grayscale value of the pixel exceeds 255, which is recorded as 255), to realize the adjustment of the overall grayscale value of the image, thereby increasing the overall brightness of the image; (d) is the result of image contrast adjustment, which calculates the average grayscale value of each pixel in the image, and adjusts the grayscale value of each pixel in a certain proportion according to the contrast ratio (set to 1.5) to achieve contrast enhancement. Although the three grayscale linear transformation methods all highlight the crack area features, none of them significantly weaken the noise information from the asphalt aggregate gap.

(2) Grayscale Nonlinear Transformation Method

**Figure 6:**
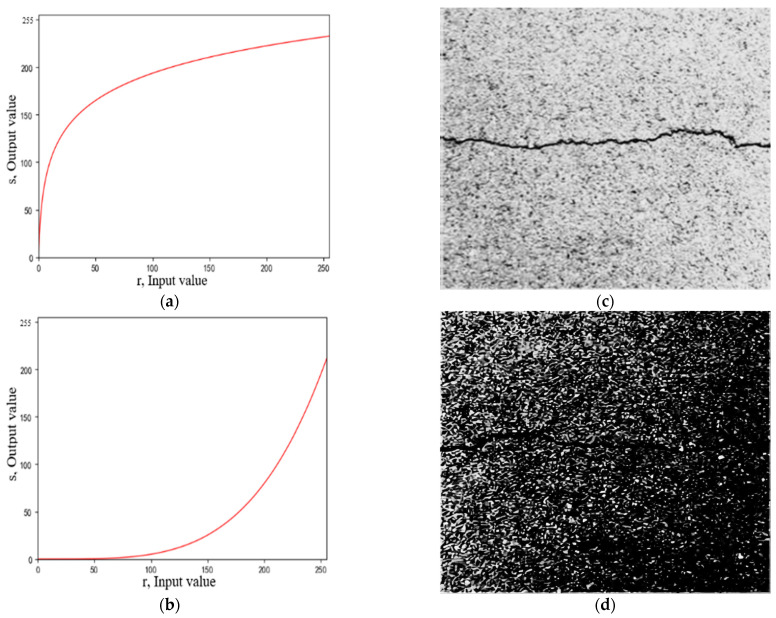
Function and results of grayscale nonlinear transformation method. (**a**) Logarithmic transformation function. (**b**) Exponential transformation function. (**c**) Logarithmic transformation result. (**d**) Gamma transformation result.

Commonly used grayscale nonlinear transformation methods include logarithmic transformation and exponential transformation (gamma transformation) (Singh and Bhandari 2020). Figure 6 shows the function images and results of these two grayscale nonlinear transformation methods. Although the logarithmic transformation method improves the contrast between the crack area and the background area, it does not significantly weaken the noise from the asphalt aggregate gap. The exponential transformation method reduces the grayscale value of the entire crack image; however, it does not highlight the features of the crack area or weaken the noise from the asphalt aggregate gap.

(3) Grayscale Segmented Linear Transformation Method

The grayscale segmented linear transformation method can enhance the region of interest and suppress the region of noninterest while improving image contrast. Equation (1) shows the segmented linear transformation function.

(1) $$S=\left\{\begin{array}{c} K_{1}\times r,\;\;\;\;\;0\leq r\leq r_{1}\\ K_{2}\times (r-r_{1})+s_{1},\;\;\;r_{1}\leq r\leq r_{2}\\ K_{3}\times \left(r-r_{2}\right)+s_{2},\;\;\;{\;r}_{2}\leq r\leq 255 \end{array}\;\;\right.$$

**Figure 7:**
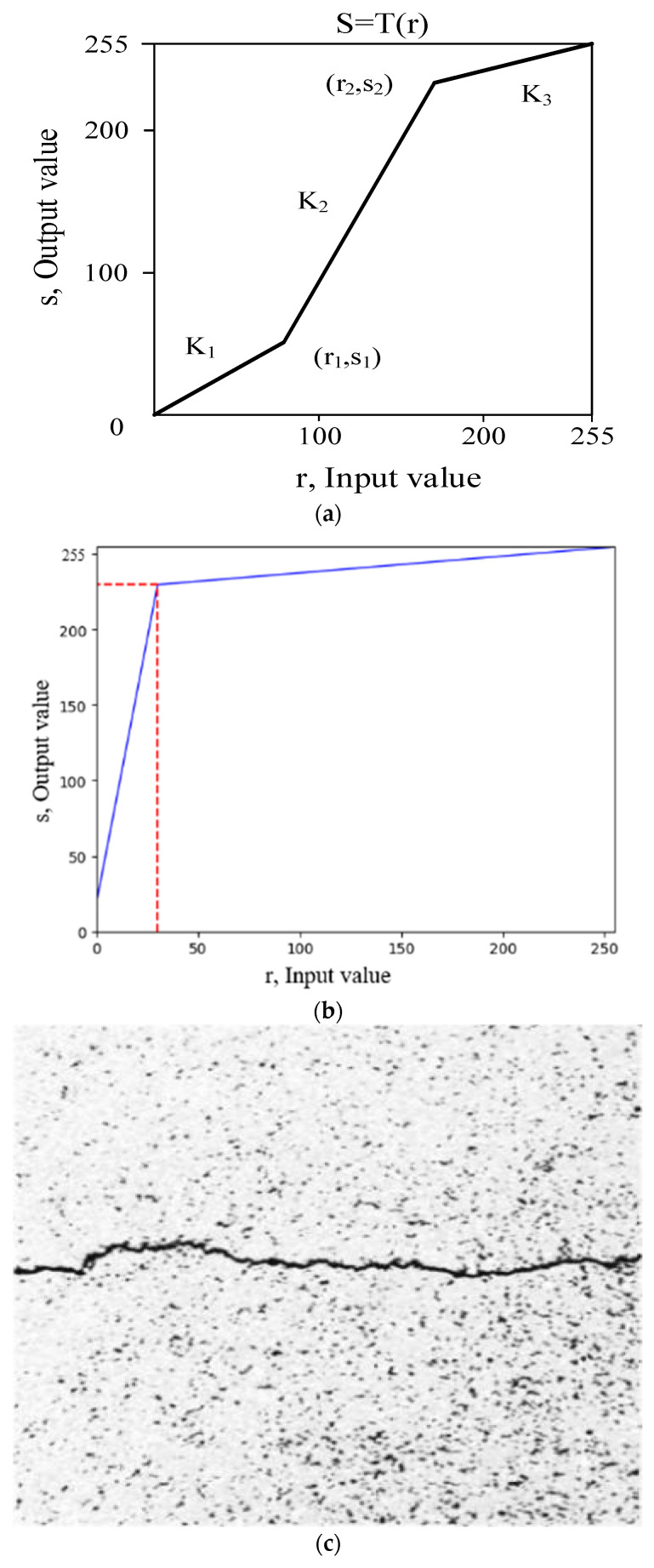
Function and results of segmented linear grayscale transformation. (**a**) Segmented linear function. (**b**) Segmented linear function used in this paper. (**c**) Results of segmented linear grayscale transformation.

Here, *S* is the output gray level and *r* is the input gray level. *K*_1_, *K*_2_, and *K*_3_ are the three polyline slopes. (*r*_1_, *s*_1_) and (*r*_2_, *s*_2_) are the two inflection point coordinates of the polyline. Figure 7a shows the grayscale segmented linear function. The image grayscale range ((0, *r*_1_), *(r*_1_, *r*_2_), (*r*_2_, 255)) can be stretched or shrunk corresponding to the slope of different polyline segments to (0, *s*_1_), (*s*_1_, *s*_2_), (*s*_2_, 255), respectively, as needed. The cracks in the asphalt pavement image are the darkest. To achieve a complete retension of their characteristics during a segmented linear grayscale transformation, the smaller values of *r*_1_ and *r*_2_ shall be selected as much as possible without losing the features of the region of interest. Therefore, through a comprehensive analysis of the image features and processing results of the crack dataset, in this paper, *r*_1_, *r*_2_, *s*_1_, and *s*_2_ are, respectively, set to 0, 30, 20, and 230. Figure 7b shows the segmented linear function used in this paper.

Figure 7c illustrates the effectiveness of segmented linear grayscale transformation in preserving the crack profile details while enhancing the crack feature information. The method also greatly weaken the noise information from the asphalt aggregate gap, thus improving the overall visual representation of the image. As a result, this paper adopts the segmented linear grayscale transformation method to process crack image from asphalt pavement after histogram equalization.

### 2.5. Image Smoothing Filter

Image smoothing and filtering calculate the average grayscale value of adjacent pixels for each pixel. The pixel is assigned the grayscale value obtained [25]. The filtering techniques include mean filtering, Gaussian filtering, and median filtering.

(1) Mean Filtering

**Figure 8:**
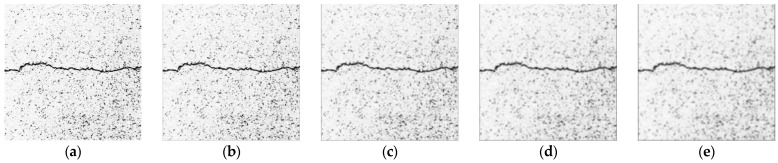
Results of mean filtering. (**a**) Original image. (**b**) 3 × 3 template. (**c**) 5 × 5 template. (**d**) 7 × 7 template. (**e**) 9 × 9 template.

Mean filtering replaces each grayscale value in the original image with the average value in the adjacent area [26]. The results of this method are shown in Figure 8. The comparison of the processing results of the mean filtering templates of different sizes shows that the larger the size of the template, the more obvious the weakness of the noise information, but also more obvious is the crack image loss with blurred details and crack edges.

(2) Gaussian Filtering

**Figure 9:**
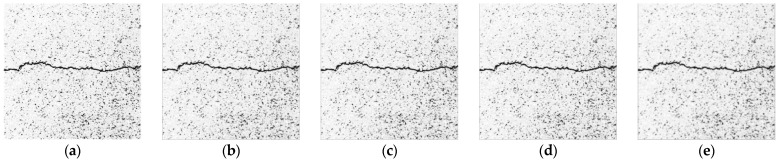
Results of Gaussian filtering. (**a**) Original image. (**b**) 3 × 3 template. (**c**) 5 × 5 template. (**d**) 7 × 7 template. (**e**) 9 × 9 template.

In the Gaussian filtering template, the weight coefficient of the element is calculated using the two-dimensional Gaussian distribution function based on the distance between the current pixel and the pixel to be processed. The point with a shorter distance from the pixel to be processed is given a larger weight, otherwise it is given a smaller weight [27]. Figure 9 shows the results of Gaussian filtering. Compared with using mean filtering, this method causes less damage to the image details and the overall image is smoother. Using different templates of Gaussian filtering does not have a significant impact on the appearance of the image. Furthermore, there are no obvious weaknesses in the noise areas of the asphalt aggregate gap.

(3) Median Filtering

**Figure 10:**
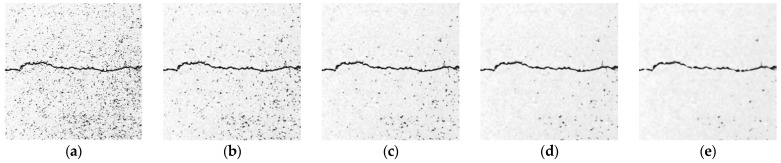
Results of median filtering. (**a**) Original image. (**b**) 3 × 3 template. (**c**) 5 × 5 template. (**d**) 7 × 7 template. (**e**) 9 × 9 template.

The median filtering method selects a window centered on each pixel point. It sorts the grayscale value of each pixel point in the window in a one-dimensional space. Then, it assigns the grayscale value of the middle sequence position to the current pixel point [28]. For images with obvious noise, since the grayscale value of the noise pixel point is significantly higher than that of the surrounding points, the grayscale value of the noise pixel will not be ranked in the middle of the sequence, and this achieves the removal of noise. Figure 10 shows the results of median filtering. By increasing the size of the filter template, median filtering becomes increasingly effective in removing the noise areas from the gaps between asphalt aggregate. When the template size is 9 × 9, most of the noise can be filtered out while preserving the crack details. However, as the size of the filter template increases, the clarity of the crack profile decreases and may even fracture.

Overall, the mean filtering method loses the detail information of the crack area while removing the noise area from the asphalt aggregate gap. Gaussian filtering causes less damage to the image details and completely retains the detailed information of crack area. But there are no obvious weaknesses in the noise area from the asphalt aggregate gap. Median filtering could finish the task of filtering the noise from asphalt aggregate gap well and could relatively hold the completely detailed information of the crack area. Based on the characteristics of the asphalt pavement crack dataset, and considering the filtering results of the noise area from the asphalt aggregate gap and the integrity of the crack details, this paper uses the median filtering template with a window size of 9 × 9 to filter the grayscale transformed image.

### 2.6. Image Binarization

Image binarization can effectively separate the crack area from the background area, and this is highly important to the segmentation and extraction of the crack area [29]. At present, the threshold segmentation method [30] is commonly used for image binarization processing. That is, when the gray value of a pixel is less than or equal to the set threshold, the gray value is set to 0. It is set to 255 when the gray value exceeds the set threshold. The threshold segmentation methods consist of the global threshold method [31] and the local threshold method [32].

(1) Global Threshold Method

If the valley between the two peaks of the crack region and the background region in the image histogram is used as the threshold, the segmentation of the target region and the background region can be achieved. However, different methods are used in different valleys, resulting in different effects on the thresholds and the processing. The simplest global threshold method is the fixed threshold method, i.e., setting the threshold T as an intermediate value (127) or customizing it as needed. However, it is difficult to determine the optimal threshold for multiple input images. The Otsu algorithm, which is widely used currently, is an adaptive threshold selection method. It sets each gray value as a threshold, and selects the gray value corresponding to the largest interclass variance as the final threshold [33]. The binarization effect is better when the contrast between the crack region and the background region is higher or the histogram presents double peaks. But when the size ratio of the crack region and the background region is large or the grey level is close, this will lead to a large gap between the peaks of the double peaks in the histogram, or even a single peak if the binarization effect is poor.

**Figure 11:**
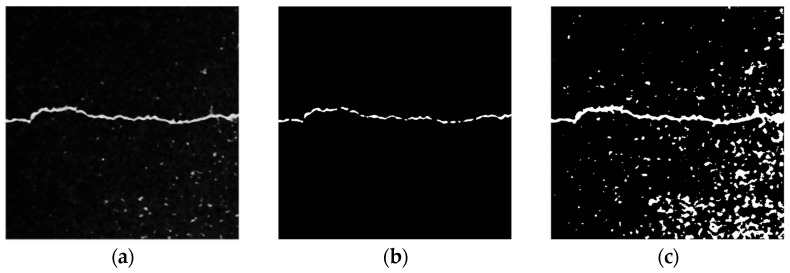
Results of global threshold method. (**a**) Original image. (**b**) Threshold T = 200. (**c**) Otsu algorithm.

Figure 11 shows the processing results for these two global threshold methods. Figure 11b displays the processing results for a larger threshold (T = 200). Although it ensures that all input images completely filter out the noise from the asphalt aggregate gap, it leads to a considerable loss of details in the crack area, and even leads to breaks. In Figure 11c, the Otsu algorithm preserves relatively complete information about the crack profile, but fails to filter out noise from the asphalt aggregate gap.

(2) Local Threshold Method

**Figure 12:**
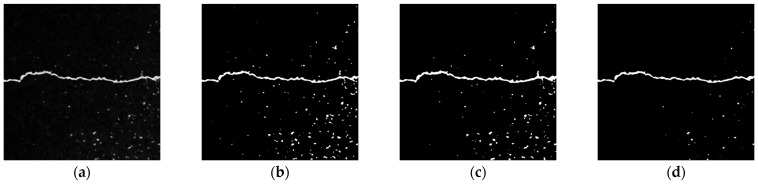
Results of local threshold method. (**a**) Original image. (**b**) Niblack algorithm. (**c**) Nick algorithm. (**d**) Sauvola algorithm.

After the histogram equalization of the crack image, the peaks corresponding to the crack area and the background area are relatively close or have a large peak difference [34]. This results in the histogram converging to a single peak. In this case, the local threshold method can be used to independently calculate the threshold based on the local information of each pixel. Currently, the commonly used local threshold methods include the Niblack [35], Nick [36], and Sauvola [37] algorithms, whose processing results are shown in Figure 12.

The binarization results of the Niblack algorithm depend on the local window; therefore, the background pixels will be misjudged as foreground pixels when they all appear as noise areas of the asphalt aggregate gap in the local window. When they all appear as crack areas in the local window, parts of the detail will be lost. There is still a lot of noise in the crack-filtered image after being processed using the Niblack algorithm, as shown in Figure 12b. According to some scholars, the Nick algorithm, which is based on the Niblack algorithm, has a better processing rate for images with low contrast or high background brightness. However, for the filtered images processed in this paper, the advantages of the Nick algorithm in processing high-brightness backgrounds cannot be brought into play. It has no obvious weakening on the asphalt aggregate gap noise, as shown in Figure 12c. The Sauvola algorithm improves the Niblack algorithm. Compared with other local threshold algorithms, the Sauvola algorithm preserves the crack area details as much as possible, and it suppresses the noise from the asphalt aggregate gap in the image, as shown in Figure 12d.

## 3. Crack Area Extraction

### 3.1. Mathematical Morphology Method

**Figure 13:**
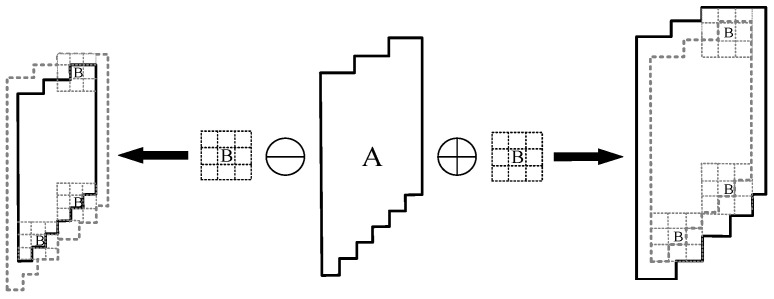
Schematic diagram of expansion and erosion operation. The arrow on the left indicates that B is used to erode A. The arrow on the right indicates that B is used to expand A.

Mathematical morphology refers to a mathematical tool [38] for analyzing images based on their morphology. The commonly used morphological operations include expansion, corrosion, opening operation (corrosion first, then expansion) [39], and closing operation (expansion first, then corrosion) [40]. Figure 13 shows a schematic diagram of an expansion and erosion operation.

Opening and closing are combined operations of expansion and corrosion. A○B is written as the opening operation of B against A. The opening operation can eliminate the burr in the crack profile and the isolated noise in the asphalt aggregate gap. This makes the crack profile even and smooth, with little effect on the crack area. A●B is written as the closed operation of B against A. The closing operation can fill the voids in the crack area, making the crack connection area complete and full. It has no obvious suppression in the noise area of asphalt aggregates.

### 3.2. Crack Area Extraction Method

The image binarization operation can filter out most of the noise from the asphalt aggregate gap. However, it is still impossible to obtain a binary image just containing the crack profile. It is also difficult to segment and extract the crack area. Therefore, based on the binary image obtained by the global threshold method and local threshold method, combined with mathematical morphology and connected domain threshold method [41], this paper proposes three methods of crack area extraction: global threshold + expansion operation, local threshold + opening operation, and local threshold + connected domain threshold.

(1) Global Threshold + Expansion Operation

**Figure 14:**
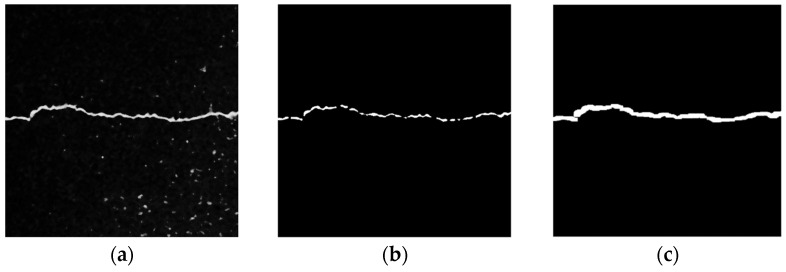
Global threshold + expansion operation. (**a**) Original image. (**b**) Global threshold *T* = 200. (**c**) Expansion operation.

Figure 14 shows the processing results of the global threshold + expansion operation. When using the global threshold method to binarize the image, a larger threshold (T = 200) is selected for all images. This operation filters out the noise from the asphalt aggregate gap in the image. However, a lot of details of the crack area will be lost, resulting in the reduction of the crack area, and sometimes breaks occur, as shown in Figure 14b. To solve this problem, the expansion operation in the mathematical morphology method is used to expand and fill the binary image of the crack profile and enrich and supplement the information about the crack area. As shown in Figure 14c, when the crack width increases, the original broken crack profile is basically connected to a connected domain.

(2) Local Threshold + Opening Operation

**Figure 15:**
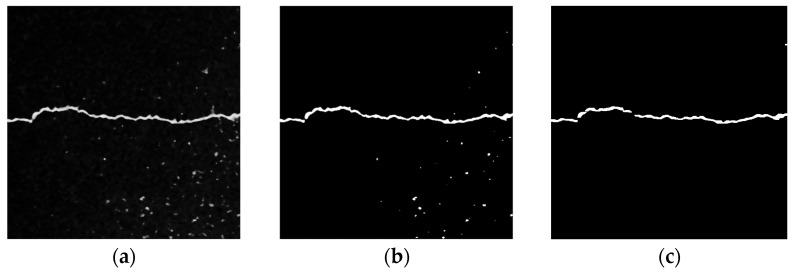
Local threshold + opening operation. (**a**) Original image. (**b**) Sauvola algorithm. (**c**) Opening operation.

Figure 15 shows the processing results of the local threshold + opening operation. Figure 15b shows the image processing results binarized using the Sauvola algorithm in the local threshold method. Although the details of the crack area are relatively complete, there are still some isolated noise points in the asphalt aggregate gap. To solve this problem, the opening operation in the mathematical morphology method is used to eliminate these noises. As shown in Figure 15c, the opening operation can eliminate the burr in the crack area and the isolated noise from the asphalt aggregate gap. It can preserve the integrity of the original crack as much as possible, but some details of the crack profile may be lost.

(3) Local Threshold + Connected Domain Threshold

**Figure 16:**
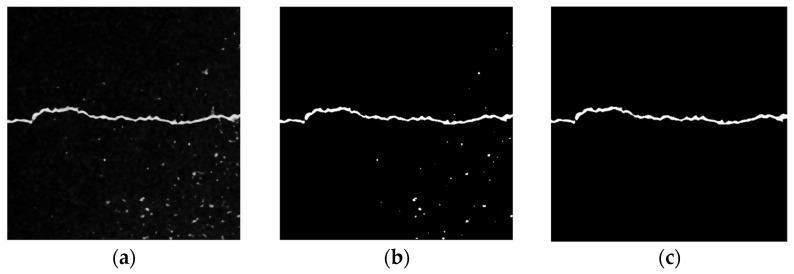
Local threshold + connected domain threshold. (**a**) Original image. (**b**) Sauvola algorithm. (**c**) Connected domain threshold.

**Figure 17:**
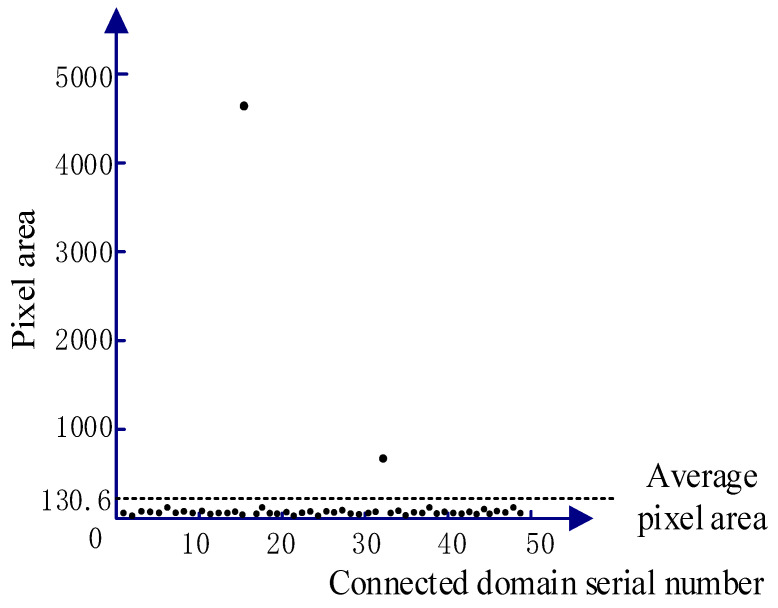
Statistics of connected domain pixel area.

Figure 15 depicts the processing results of the local threshold + connected domain threshold. The Sauvola algorithm in the local threshold method binarizes the image. As shown in Figure 16b, the details of the crack area are basically preserved, but there are still some noises from the asphalt aggregate gap. Due to the small gap area of the asphalt aggregate, the noise pixel area is also relatively small. As illustrated in Figure 16b, Figure 17 shows the statistical results of the pixel area of each connected domain. There are 48 connected domains (white areas) in the image after binary processing by the Sauvola algorithm. The average pixel area of each connected domain is 130.6, of which the pixel area of two connected domains is greater than the average value, and the pixel area of the remaining 46 connected domains is lower than the average value. Therefore, based on the large area gap between the crack area and the noise pixel of asphalt aggregate gap, the connected domain threshold method is used to filter the connected domain whose pixel area is lower than the average value or the preset threshold value, so as to extract the main crack area. As shown in Figure 16c, this method can not only fully preserve the details of the crack area, but also completely filter out the noise area.

### 3.3. Evaluations on Crack Extraction by Three Methods

**Figure 18:**
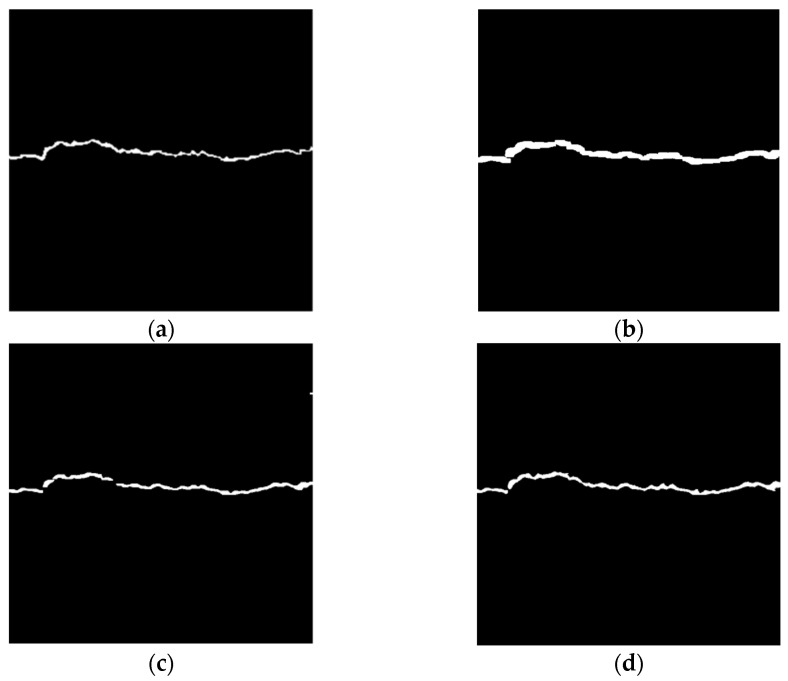
Comparison of results of various methods. (**a**) Manual pixel calibration. (**b**) Global threshold + expansion operation. (**c**) Local threshold + opening operation. (**d**) Local threshold + connected domain threshold.

Figure 18 shows the crack extraction results of global threshold + expansion operation, local threshold + opening operation, and local threshold + connected domain threshold. In the figure, (a) is a binary image for artificial pixel calibration, which is almost identical to the overall profile and pixel area of the crack area in the original image, and (a) can be used as a reference for the above three methods; (b), (c), and (d) are the results of the above three methods, respectively. All the three methods can filter out the noise area from asphalt aggregate gaps. However, it is a challenge to accurately evaluate the integrity of the details of the crack area only through visual inspection. To meet the accuracy requirements of the calculation of the pixel area of the subsequent crack area, this paper evaluates the extraction results of all the three crack extraction methods from the aspects of the image matching degree and the error of the pixel area of the crack area.

➀ Evaluation on Image Matching Degree

It is used to evaluate the profile shape and texture direction of the cracks processed using the three methods based on the image matching degree. The first step is to randomly select 100 images from self-collected asphalt pavement crack images for artificial pixel calibration. The second step is to extract the crack area using the above three methods. The final step is to evaluate the artificial-pixel-calibrated images on their matching degrees with the images processed by the above three methods. Assuming that the artificial pixel calibration image is *A* (*m*, *n*), and the crack extraction image is *B* (*m*, *n*), the calculation of the matching degree is shown as Equation (2).

(2) $$P=\frac{\sum_{i=1}^{m}\sum_{j=1}^{n}\left[A\left(m,n\right)\times B(m,n)\right]}{\sum_{i=1}^{m}\sum_{j=1}^{n}A(m,n)}$$

Table 1 shows the calculated matching degree. The matching degree of Method 1 is low; the extraction of cracks is poor. However, the matching degrees of Methods 2 and 3 are high, indicating that these two methods can better complete crack segmentation and extraction. Method 3 has the highest matching degree, reaching 95.24%. It proves that the image processed by Method 3 has the highest matching degree with the standard image crack area. This is coupled with good extraction, which ensures the accuracy in subsequent calculations of these crack parameters.

➁ Evaluation on Pixel Area of Crack Area

Based on the above 100 asphalt images of pavement cracks, we calculated the differences between the pixel areas of each manually calibrated image and the pixel areas of the crack area extracted by the above three methods. The difference in calculation is the error of the three crack area extraction methods. Table 2 shows the results.

Table 2 shows that the average error of the crack extraction pixel area of Method 1 for 100 images is +1024. This accounts for 18.62% of the manually calibrated pixel area, whose error is too large to meet the accuracy of the detection requirements. The average pixel area errors of Method 2 and Method 3 are +556 and +348, respectively. These errors account for 10.11% and 6.33% of the artificially calibrated pixel area, whose errors are relatively small and can meet detection requirements. Among them, the processing results of the opening operation in Method 2 are different for each noise information and each crack information in each image. These results show that the pixel area error variance is large, and the crack area extraction effect is unstable. In Method 3, the connected domain threshold method only filters out the noise area and has almost no effect on the crack profile and crack pixel area. This shows that the variance of the pixel area error is small with relatively stable crack area extraction.

**Figure 19:**
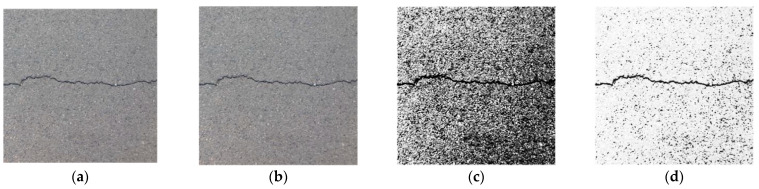

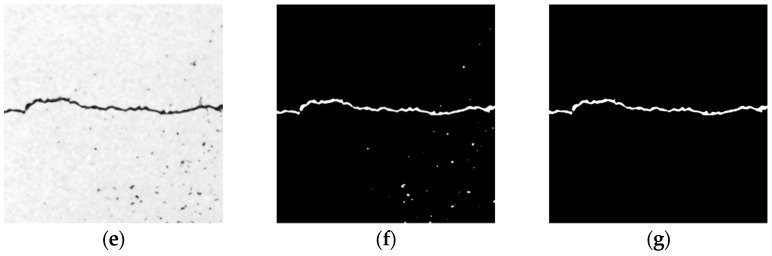
Example of image processing. (**a**) Original image. (**b**) Grayscale processing. (**c**) Histogram equalization. (**d**) Segmented linear transformation. (**e**) 9 × 9 median filtering. (**f**) Sauvola binarization. (**g**) Connected domain threshold.

Based on the evaluation results of the matching degree and crack pixel area, this paper selects Method 3 (local threshold + connected domain threshold) as the crack area segmentation and extraction method, because it is better and has more stable crack area extraction. Figure 19 shows an example of the extraction results.

In Figure 19, (a) is the original image; (b) is the result of image grayscale processing, which deletes the color information, reduces image parameters, and improves processing speed; (c) is the result of image histogram equalization, which improves the image contrast and highlights the detailed features of cracks; (d) is the result of segmented linear grayscale transformation, which further improves the image contrast and clarity, and enhances the grayscale detail of the region of interest and suppresses gray levels of the region of no interest; (e) is the image after the median filtering with a template size of 9 × 9, which filters out most of the noise of asphalt aggregate gap while preserving the crack details; (f) is the image binarized by the Sauvola algorithm, which converts the image to a binary image that can further enhance the image contrast and improve the image processing speed while filtering out the noise area; (g) is the result of the connected domain threshold method, which filters out all the noise and completes the segmentation and extraction of the crack area without affecting the details of the crack area.

## 4. Calculation of Actual Crack Parameters

### 4.1. Calculation of Actual Crack Area

In this paper, the actual crack area is calculated through the pixel area of the crack extraction area, as shown in Equation (3).

(3) $$\frac{S}{s}=\beta \;$$

Here, *S* is the actual area of the crack, *s* is the pixel area of the crack, and *β* is the magnification between the pixel area and the actual area. When the crack pixel area *s* is given, only the magnification *β* is required to obtain the actual crack area.

**Figure 20:**
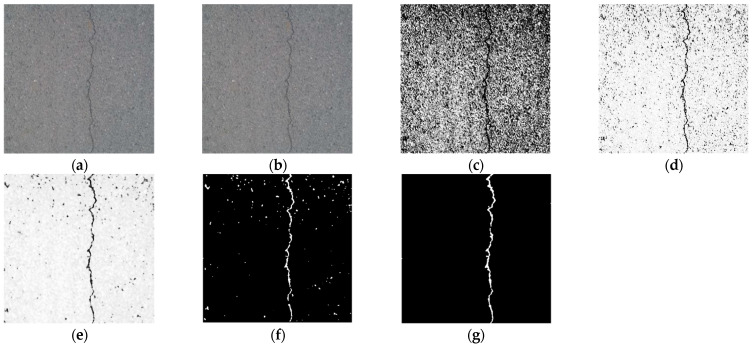
Extraction of crack area. (**a**) Original image. (**b**) Grayscale processing. (**c**) Histogram equalization. (**d**) Segmented linear transformation. (**e**) 9 × 9 median filtering. (**f**) Sauvola binarization. (**g**) Connected domain threshold.

**Figure 21:**
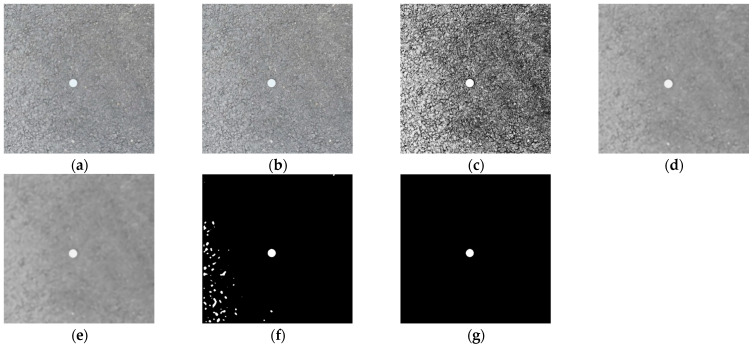
Extraction of reference pixel area. (**a**) Original image. (**b**) Grayscale processing. (**c**) Histogram equalization. (**d**) Segmented linear transformation. (**e**) 9 × 9 median filtering. (**f**) Sauvola binarization. (**g**) Connected domain threshold.

This paper uses the calibration object method to calculate the magnification *β* [42]. The principle is that the image magnification *β* is also fixed with the distance between the camera and the road surface. The shooting angle and focal length are also fixed. Therefore, a calibration object with high precision and a known actual area can be used as a reference. The captured image is segmented to extract the pixel profile and calculate the pixel area of the calibration object. This information is then used to derive the image magnification *β*. An example of this method is seen as follows:

(1) Fix the camera at a certain height on the surface of the road. Take a standard table tennis ball with the same plane area at each angle as a reference and place it on the ground. Capture crack images and table tennis ball images, respectively.
(2) Extract the crack pixel area from the captured image according to the image enhancement method proposed in this paper. The extraction results are shown in Figure 20, and the pixel area of the crack is calculated as *s*_1_ = 4543 pixels.

(3) Calculate the actual plane area of a standard table tennis ball with a diameter of 40 mm *S*_2_ = 3.14 × 20^2^ = 1256 mm^2^, and perform pixel region segmentation and extraction on the captured image of the table tennis ball. The extraction results are shown in Figure 21.

(4) Calculate the pixel area of the table tennis ball in the captured image as *s*_2_ = 908 pixels; the magnification at this time can be calculated as follows:

(4) $$\beta =\frac{S_{2}}{s_{2}}=\frac{1256}{908}=1.38\;{\mathrm{mm}}^{2}/\mathrm{pixels}\;$$

(5) Calculate the actual area *S*_1_ of the crack through the pixel area *s*_1_ of the crack area and the magnification *β*:

(5) $$S_{1}=s_{1}\times \beta =4543\times 1.38=6284.14\;{\mathrm{mm}}^{2}$$

For block cracks, tortoise cracks, and other mesh cracks, the impact area of the crack is the minimum outer rectangular area of the crack, which is the same as the area of the selected area of the crack positioning anchor box in this paper. Therefore, the calculation of the pixel area of mesh cracks can be converted into the calculation of the pixel area of the selected area of the mesh crack anchor box, and then the actual impact area is calculated based on the magnification ratio. For transverse and longitudinal cracks, the impact area is calculated by multiplying the length of the crack by 0.2 m. Therefore, the impact area of strip cracks needs to be calculated based on the extraction of the contour of the crack area and the corresponding physical parameters of the crack.

### 4.2. Calculation of Actual Crack Width

**Figure 22:**
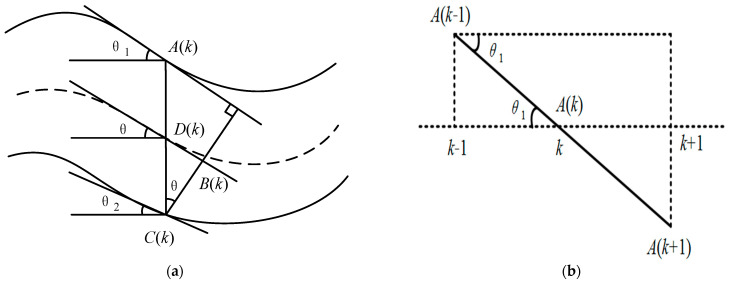
Calculation of crack width. (**a**) Pixels in Column *k* of the crack. (**b**) Pixels on the upper edge of the crack.

For cracks extracted from binary images, we traverse each point in the image with a loop statement. We express all pixels as (*i*, *k*), where i represents the number of pixel rows (*i* = 1, 2, 3, …), and k represents the number of pixel columns (*k* = 1, 2, 3, …). The specific steps to calculate the crack width are as follows:

➀ Figure 22 shows the calculation of crack width. After traversing Column *k* of pixels and determining the row numbers *A*(*k*) and *C*(*k*) corresponding to the pixels at the upper and lower edges of the crack in this column, the number of pixel rows *D*(*k*) corresponding to crack width in this column can be calculated, as shown in Equation (6).

(6) D(k)=|A(k) −C(k)| 

➁ Figure 22b shows the pixels on the upper edge of the crack. The upper edge of the crack corresponds to the number of rows *A*(*k +* 1) and *A*(*k* − 1) in Column *k +* 1 and Column *k* − 1, respectively, and the calculation for the inclination angle $\theta_{1}$ corresponding to the upper edge of the pixel crack in Column *k* is as shown in Equations (7) and (8).

(7) $$\tan\theta_{1}=\frac{\left|A(k-1)\;-A(k+1)\right|}{(k+1)-(k-1)}\;\;\;$$

(8) $$\theta_{1}=\arctan[\frac{\left|A\left(k-1\right)\;-A(k+1)\right|}{2}]\;$$

Similarly, it can be obtained that the angle of inclination $\theta_{2}$ corresponds to the lower edge of the pixel crack in Column *k*, as shown in Equation (9).

(9) $$\theta_{2}=\arctan[\frac{\left|C\left(k-1\right)\;-C(k+1)\right|}{2}]\;\;$$

➂ From the angles of inclination $\theta_{1}\;and\;\theta_{2}$ that correspond to the upper and lower edges of the pixel crack in Column *k*, it is possible to approximate the angle of inclination of the central axis of the crack θ:

(10) $$\theta =\frac{\theta_{1}+\theta_{2}}{2}\;\;\;\;\;\;\;$$

At this time, the overall angle of inclination of the crack at Column *k* of pixels can be approximated as θ, $\theta_{1}=\theta_{2}=\theta$.

➃ The pixel crack width *B*(*k*) in Column *k* can be approximated as follows:

(11) B(k)=D(k)×cosθ

➄ We traverse each pixel column in the image, and calculate and record the crack widths *B*(1), *B*(1), *B*(1), …, *B*(*k*) of different columns of pixels, and the average crack width *B* is shown as Equation (12).
  (12) $$B=\frac{B\left(1\right)+B\left(2\right)+B\left(3\right)+\ldots +B(k)}{k}\;$$

### 4.3. Calculation of Actual Crack Length

The actual crack area *S* and the average crack width *B* are calculated by the above method. Equation (13) approximates the actual crack length L. This method is simple, fast, and direct. However, due to the superposition of various numerical errors, the calculated results of crack lengths in some images may greatly differ from the actual measured values whose accuracy fails to meet the requirements. This paper adopts the crack skeleton extraction method to calculate the crack length L, which mainly includes crack skeleton extraction and burr removal.

(13) $$L=\frac{S}{B}\;$$

(1) Extraction of Crack Skeleton

The crack skeleton is used to compress the crack width to a single pixel width and remove the redundant profile feature information of the crack, so that the direction and topological structure of the crack can be displayed directly and clearly. Among the commonly used skeleton extraction methods, the Hilditch algorithm is slower and more challenging to process. The Rosenfeld algorithm [43] calculates rapidly but it is prone to cracks and breaks. The Zhang–Suen iterative thinning algorithm is relatively simple and fast and preserves image foreground features. Therefore, in this paper, the Zhang–Suen algorithm is used to extract the crack skeleton of an asphalt pavement.

The binary image used in this paper has a pixel value of 255 for the crack region and 0 for the background. For a pixel P_1_, the pixel directly above it will be noted as P_2_, following the concept of eight neighbors. Other surrounding pixels are recorded as P_3_, P_4_, … and P_9_ clockwise [44]. The Zhang–Suen iterative refinement algorithm [45] traverses all crack pixels P_1_ to determine whether the pixel meets the following conditions:

➀ Among the eight neighboring pixels of P_1_, the number *N* of crack pixels is 2 ≤ *N* ≤ 6;
➁ In the clockwise sorting of the eight neighboring pixels of P_1_ (P_2_, P_3_, …, P_9_, P_2_), the pixel value of the adjacent pixel changes from 0 to 255 only once;
➂ There is at least one background pixel in P_2_, P_4_, and P_6_, and there is at least one background pixel in P_4_, P_6_, and P_8_;
➃ There is at least one background pixel in P_2_, P_4_, and P_8_, and there is at least one background pixel in P_2_, P_6_, and P_8_.

**Figure 23:**
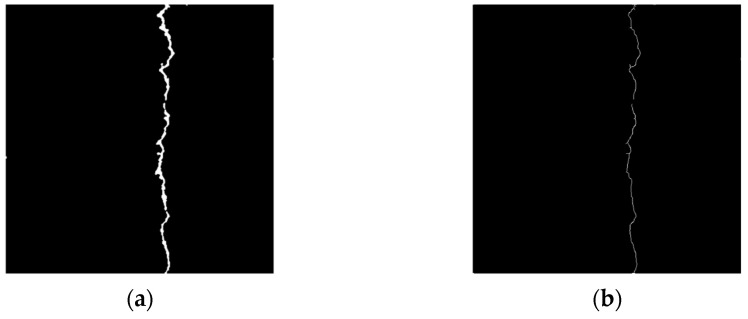
Results of crack skeleton extraction images using the Zhang–Suen algorithm. (**a**) Crack binary image. (**b**) Crack skeleton extraction image.

We select the crack pixels satisfying Condition 1, 2, and 3 and the crack pixels satisfying Condition 1, 2, and 4. We convert these two types of pixels into background pixels. Figure 23 shows the results of crack skeleton extraction.

(2) Removal of Crack Skeleton Burrs

**Figure 24:**
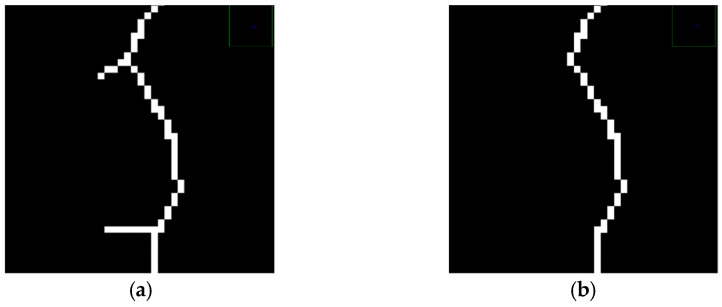
Removal of burrs from crack skeleton. (**a**) Details of burrs in crack skeleton. (**b**) Removal of burrs from crack skeleton.

For images of crack skeleton extraction, there are still many crack burrs, as shown in Figure 24a. In order to avoid the impact on the calculation of the subsequent crack length, this paper uses the connected domain threshold method to remove crack skeleton burrs based on the concept of eight neighbors. The specific steps are as follows:

➀ Traverse all crack pixels in P_1_ and count the number of pixels in the eight neighbors of P_1_. If the number is greater than 2, record P_1_ and convert the pixel value to 0.
➁ After P_1_ is converted into the background area, there will be many crack branch-connected domains. Calculate the number of pixels in each connected domain separately.
➂ Set an appropriate threshold and delete the connected domains whose number of pixels is lower than the threshold.
➃ Traverse all the pixels converted to the background area. If the number of crack pixels in the eight neighbors of pixel P_1_ is greater than 1, restore the pixel value from 0 to 255.

Figure 24b shows the result of the connected domain threshold method processing. It takes advantage of the fact that there are fewer pixels in the connected domain of the crack burr branch. It can effectively remove the crack burr and the isolated noise points omitted after distinguishing the connected domain. The crack skeleton binary image is traversed after burr removal. The crack pixel length l represents the number of cracks detected at present. The actual crack length *L* is as shown in Equation (14).

(14) L=l×β   

### 4.4. Calculation of Segments of Map Cracking

For block, tortoise, and other network cracks, the degree of damage should be judged not only with reference to the width of the crack, but also to the degree of the crack block, and the steps of calculating the degree of the crack block are as follows:

➀ Extract the crack area from the massive crack and craze crack image to obtain the crack skeleton image.
➁ Traverse all the pixels of the crack skeleton image. Count all the pixels (*x*, *y*) in Column *j*, and subtract each of the values of vertical coordinates *y*_1_, *y*_2_, *y*_3_… of the adjacent pixels in Column *j* from any of the other values of the same to obtain the difference *H*_1_, *H*_2_, *H*_3_*…* between adjacent ordinates.
➂ Similarly, count all the pixels (*x*, *y*) in Row *i*, and subtract each of the values of the abscissas *x*_1_, *x*_2_, *x*_3_… of adjacent pixels in Row *i* from any of the other values of the same to obtain the difference *W*_1_, *W*_2_, *W*_3_… between adjacent abscissas.
➃ Obtain the maximum value among *H (H*_1_, *H*_2_, *H*_3_…) and *W (W*_1_, *W*_2_, *W*_3_…), and record it as the crack segment *k*, i.e., k=max(maxH,maxW).
➄ *k* is the pixel segment of the map cracks, and the actual segment *K* of the crack is shown in Equation (15).
  (15) K=k×β  

## 5. Verification of Crack Parameter Calculation Method

At present, some scholars have carried out research on road crack detection based on two-stage target detection model and one-stage target detection model, respectively. Sekar et al. proposed a new method of road crack detection based on Faster-RCNN, and the experimental results show that the detection accuracy of this method on a homemade dataset is better than that of existing methods [46]; Su et al. proposed a method using street view images based on YOLOv5. The algorithm gives some weights to the improved loss function, which improves the feature extraction ability of the model on the details of the cracks [47]; Shu et al. proposed a method based on YOLOv5 to collect pavement cracks using street view images, and achieved better results on homemade datasets [14]; Han et al. proposed a method based on SSD for automatic pavement crack detection, which can be applied to the original images without undergoing any preprocessing, and it still had a good detection effect [48].

Although the model of two-stage target detection has high detection accuracy, the detection speed is slow, which makes it difficult to meet the real-time detection requirements. The one-stage target detection model greatly improves the target detection speed, but the detection accuracy still has a certain gap compared with the two-stage target detection algorithm. By contrast, the manual labeling method can better calculate the crack area under the premise of ensuring the accuracy.

In order to verify the accuracy of the method of crack parameter calculation in this paper, the camera was set up at a fixed height on one side of the car, and the shooting angle and focal length were kept unchanged, randomly shooting 10 cracks of different lengths. We used the method in this paper to calculate the length, width, and area of cracks, and compared them with the field measurement of cracks results. Since the actual crack area cannot be obtained by field measurement, this paper performs artificial pixel calibration on the collected images and compares their pixel area with the pixel area calculated by the method in this paper to verify the crack area calculation accuracy. The comparison results are shown in Table 3.

Table 3 shows that the relative errors of the crack length and crack pixel area are both lower than 10%, and this indicates that the calculation method for crack parameters in this paper can calculate crack length and area accurately. In the actual measurement of crack length, some tortuous cracks cannot be accurately measured with tape, and can only be approximated by the length of a straight line, resulting in the actual measured length of the crack being smaller than the length obtained by the method in this paper, which increases the relative error. Therefore, the relative error in the case of accurate measurement of the actual crack length shall be lower than the values in Table 3. That is to say, the calculation method of crack length in this paper is better than the effect reflected in the table. In Table 3, the relative errors in calculating the widths of No. 3, 6, and 9 cracks are large. On the one hand, No. 3, 6, and 9 cracks are all less than 5 mm. Since the crack width is measured with a vernier caliper, the smaller the width, the harder it is to measure. The relative error is also larger. On the other hand, according to the “Highway Technical Condition Evaluation Standard (JTG 5210-2018)” [49], the smaller the crack width, the lower the corresponding damage, and the impact on the calculation of the overall pavement crack damage rate is also small. Consequently, it is believed that the method for calculating crack width in this paper can accurately detect cracks within the allowable error range. Compared with the existing research, the detection method proposed in this paper ensures the accuracy of crack detection while having a more stable operating environment, which can be better applied to the actual detection work.

## 6. Case Study

**Figure 25:**
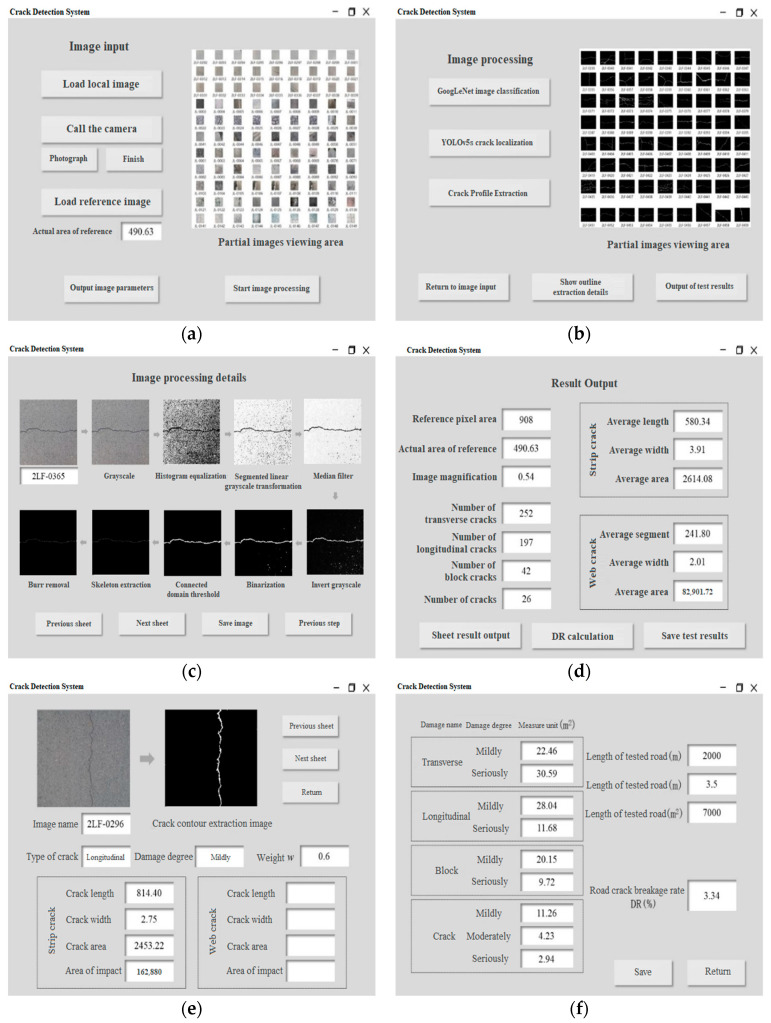
Crack detection system application examples. (**a**) Load local image. (**b**) Crack profile extraction. (**c**) Image processing details. (**d**) Overall test result output. (**e**) Single image detection result output. (**f**) Calculation of pavement crack damage rate.

On the basis of the above research, the windowing system of asphalt pavement crack detection—WSPCD1.0—is developed, which integrates and calls upon the previous research results to automatically carry out the segmentation and extraction of asphalt pavement cracks and output the physical parameters of the cracks. The specific process is as follows:

Select a road section of Xincun West Road, Zhangdian District, Zibo City, Shandong Province. According to the corresponding requirements for pavement crack image shooting, the acquired images will be detected into the pavement crack detection system, and the test interface is shown in Figure 25.

Figure 25a illustrates the loading of the local image for the image detection input, along with the importing of the reference image and manual inputting of its reference area of 490.63 mm^2^. The image browsing area depicted in Figure 25b displays the crack binary maps obtained after the crack contour extraction process. Figure 25c illustrates the image processing detail interface, which reveals the process diagram of each image as it undergoes various stages of image processing. This interface allows for browsing and saving. Figure 25d presents the output interface displaying the complete detection results of the crack image. Firstly, the image magnification *β* is calculated as 0.54 mm^2^/pixel through the pixel area of the reference and the actual area. Secondly, a total of 252 transverse cracks, 197 longitudinal cracks, 42 block cracks, and 26 cracks were counted in the images to be detected. Lastly, it was determined that the average length of the block cracks was 580.34 mm, with an average width of 3.91 mm and an average area of 2614.08 mm^2^. Additionally, the average block size of the mesh cracks was 241.80 mm, with an average width of 2.01 mm and an average area of 82,901.72 mm^2^. Figure 25e depicts the results interface for a single crack image detection. Firstly, the image browsing area displays both the original image of each crack and the extracted image of each crack. Secondly, the crack type is determined. If the crack is a strip, the system displays parameters including length, width, area, and impact area. If the crack is a mesh, the system displays parameters including blockiness, width, area, and impact area. The system assesses the degree of damage to the cracks based on present values and outputs the calculated weights. Figure 25f illustrates the interface for calculating the damage rate of pavement cracks. The interface shows statistics on the left side, indicating the impact area of each type of crack at different levels of damage. On the right side of the interface, users can manually enter the length and width of the test section, with dimensions of 2000 m and 3.5 m, respectively, to obtain the total detection area of the road segment. The pavement crack damage rate (DR) can be calculated using Equation (16) [50], which yields a result of 3.34%.

(16) $$\mathrm{DR}=100\times \frac{\sum_{i=1}^{i_{0}}w_{i}A_{i}}{A}\;\;\;$$

where *A* is the area of the detected road section, *A_i_* is the total area of pavement crack damage in category *i*, *i*_0_ is the number of crack damage types, *i* is the type of pavement crack damage, and *w_i_* is the weight conversion factor for pavement crack damage in category *i* [51].

## 7. Conclusions

This study proposes a method for segmenting, extracting, and calculating parameters of asphalt pavement cracks based on image processing. Conventional techniques of digital image processing (e.g., MSR and MSRCR) focus on adjusting the color or brightness of an image, while neglecting the denoising of the image. To solve this problem, this paper deleted the color information and reduced image parameters using image grayscale. It improved the image contrast while highlighting the detailed features of cracks through image histogram equalization, and further improved the contrast and clarity of cracks through a segmented linear grayscale transformation. This paper also filtered out most of the asphalt aggregate interstitial noise while retaining crack details through median filtering and converted the image to a binary image while filtering part of the noise area, further enhancing the image contrast and improving image processing speed through the Sauvola algorithm for binarization. For the crack extraction image, this paper used the Zhang–Suen thinning algorithm to extract the crack skeleton, used the connected domain threshold method to remove the crack skeleton burr, and then calculated the parameters of crack area, width, length, segments, etc., through the crack extraction image and the crack skeleton image. The method significantly reduces the amount of computation and improves the efficiency. The main contributions of this study are as follows:

(1) In this paper, spatial-domain-based image enhancement methods, including image grey scale, histogram equalization, image grey scale transformation, image smoothing filtering, and image binarization, were used to filter out all the noises without affecting the details of the crack region, so as to complete the segmentation and extraction of the crack region.
(2) Three crack region extraction methods based on crack binarized images were introduced, and the method of local thresholding + connectivity domain thresholding was identified by analyzing and evaluating both the crack coincidence and pixel area error.
(3) We proposed to calculate the actual feature parameters of the cracks, including the crack impact area, width, length, and blockiness, through crack skeleton extraction and burr removal. The accuracy of the calculation method of crack parameter in this paper was verified by comparative tests. It turned out that the asphalt pavement crack segmentation extraction method and parameter calculation method proposed in this paper can contribute to crack detection.
(4) Aiming at the lack of engineering applicability of the current asphalt pavement crack detection methods based on deep learning and image processing, and the difficulty of providing direct help for the actual detection of asphalt pavement cracks, a window system was developed. Additionally, the window system integrated and called upon the previous research results, which provided direct and accurate information of the crack parameters for the detection of cracks in asphalt pavements.

## Figures and Tables

**Table 1:** Evaluation results of matching degree.

Method	Crack Extraction	Average Matching Degree/%
Method One	Global threshold + expansion operation	81.72
Method Two	Local threshold + opening operation	91.08
Method Three	Local threshold + connected domain threshold	95.24

**Table 2:** Evaluation results of pixel area.

Method	Crack Extraction	Average Pixel Error	Pixel Error Ratio	ANOVA
Method One	Global threshold + expansion operation	+1024	18.62%	196.13
Method Two	Local threshold + opening operation	+556	10.11%	116.60
Method Three	Local threshold + connected domain threshold	+348	6.33%	42.08

**Table 3:** Comparison of calculation method in this paper and manual calculation.

Crack No.	Crack Length			Crack Width			Crack Area		
	Calculated Length/m	Actual Measured Length/m	Relative Error/%	Calculated Width/mm	Actual Measured Width/mm	Relative Error/%	Pixel Area/Pixel	Manually Calibrate Pixel Area/Pixel	Relative Error/%
1	0.89	0.85	4.99%	8.65	8.49	1.88%	6021	5816	3.52%
2	0.75	0.73	2.80%	7.19	7.29	−1.37%	5691	5464	4.15%
3	0.47	0.45	3.02%	4.32	4.62	−6.49%	3021	3254	−7.16%
4	0.92	0.87	5.68%	9.18	9.05	1.44%	6241	6094	2.41%
5	0.91	0.86	5.02%	8.18	8.36	−2.03%	5874	5716	2.76%
6	0.86	0.82	3.86%	4.64	4.28	8.41%	4452	4681	−4.89%
7	0.72	0.70	3.78%	7.19	7.33	−1.91%	5297	5014	5.64%
8	0.80	0.79	2.47%	6.19	6.35	−2.52%	5516	5364	2.83%
9	0.65	0.63	3.79%	3.26	3.69	−11.65%	4625	5021	−7.89%
10	0.60	0.58	2.80%	7.17	7.41	−3.23%	5464	5534	−1.26%

## Data Availability

The data presented in this study are available on request from the first or corresponding author.
